# Novel Neutralizing Epitope of PEDV S1 Protein Identified by IgM Monoclonal Antibody

**DOI:** 10.3390/v14010125

**Published:** 2022-01-11

**Authors:** Techit Thavorasak, Monrat Chulanetra, Kittirat Glab-ampai, Karsidete Teeranitayatarn, Thaweesak Songserm, Rungrueang Yodsheewan, Nawannaporn Sae-lim, Porntippa Lekcharoensuk, Nitat Sookrung, Wanpen Chaicumpa

**Affiliations:** 1 Graduate Program in Immunology, Department of Immunology, Faculty of Medicine Siriraj Hospital, Mahidol University, Bangkok 10700, Thailand; techit.tha@student.mahidol.edu; 2 Center of Research Excellence on Therapeutic Proteins and Antibody Engineering, Department of Parasitology, Faculty of Medicine Siriraj Hospital, Mahidol University, Bangkok 10700, Thailand; monrat.chl@mahidol.ac.th (M.C.); kittirat.gla@mahidol.edu (K.G.-a.); nawannaporn.sae@mahidol.edu (N.S.-l.); 3 MORENA Solution Company, Wang-thong-lang District, Bangkok 10310, Thailand; karsidete.t@gmail.com; 4 Department of Veterinary Pathology, Faculty of Veterinary Medicine, Kam-paeng-san Campus, Kasetsart University, Nakhon-pathom 73140, Thailand; fvetss@ku.ac.th (T.S.); fvetrry@ku.ac.th (R.Y.); 5 Department of Microbiology and Immunology, Faculty of Veterinary Medicine, Kasetsart University, Bangkok 10900, Thailand; fvetptn@ku.ac.th; 6 Biomedical Research Incubation Unit, Department of Research, Faculty of Medicine Siriraj Hospital, Mahidol University, Bangkok 10700, Thailand; nitat.soo@mahidol.ac.th

**Keywords:** porcine epidemic diarrhea virus (PEDV), spike (S) protein, monoclonal antibody, neutralization assay, neutralizing antibody, phage mimotope

## Abstract

Porcine epidemic diarrhea virus (PEDV) causes devastating enteric disease that inflicts huge economic damage on the swine industry worldwide. A safe and highly effective PEDV vaccine that contains only the virus-neutralizing epitopes (not enhancing epitope), as well as a ready-to-use PEDV neutralizing antibody for the passive immunization of PEDV vulnerable piglets (during the first week of life) are needed, particularly for PEDV-endemic farms. In this study, we generated monoclonal antibodies (mAbs) to the recombinant S1 domain of PEDV spike (S) protein and tested their PEDV neutralizing activity by CPE-reduction assay. The mAb secreted by one hybrodoma clone (A3), that also bound to the native S1 counterpart from PEDV-infected cells (tested by combined co-immunoprecipitation and Western blotting), neutralized PEDV infectivity. Epitope of the neutralizing mAb (mAbA3) locates in the S1A subdomain of the spike protein, as identified by phage mimotope search and multiple sequence alignment, and peptide binding-ELISA. The newly identified epitope is shared by PEDV G1 and G2 strains and other alphacoronaviruses. In summary, mAbA3 may be useful as a ready-to-use antibody for passive immunization of PEDV-susceptible piglets, while the novel neutralizing epitope, together with other, previously known protective epitopes, have potential as an immunogenic cocktail for a safe, next-generation PEDV vaccine.

## 1. Introduction

Porcine epidemic diarrhea virus (PEDV) is a causative agent of a highly contagious diarrheal disease in pigs, named porcine epidemic diarrhea (PED). The disease is characterized by enteritis, watery diarrhea, and vomiting, which eventually result in severe dehydration and electrolyte loss [1]. Since its discovery in 1977 in the United Kingdom [2,3], the disease has spread globally, having a devastating impact on the pig industry worldwide, particularly in North America, Europe, and Asia [4,5,6]. The PED mortality rate among piglets during the first week of life can reach 100% [4,7]. Most pigs become resistant to the disease as they grow older [8,9]. Nevertheless, PED-recovered pigs have poor growth rates, i.e., their weight accruement is retarded [4,10]. Gilts and sows infected with PEDV have poor reproductive performance from irregular/delayed estrous cycles. Additionally, they may have mastitis and agalactia, which affects the colostrum and milk feeding, as well as posing a high risk of PEDV infection to their offspring during farrowing [4,11].

PEDV uses the class I transmembrane spike glycoprotein (S) that appears as homotrimers on the virion surface to bind to the host cell receptor, i.e., porcine amino peptidase N (pAPN) on the pig enterocyte to enter the cell. Molecularly, the PEDV S protein consists of four portions, i.e., two ectosubunits, S1 and S2, with different functional activities; a transmembrane portion (TM); and a short intracytoplasmic tail (IC). The S1 functions as receptor-binding domain (RBD). It contains S1-N-terminal domain (S1-NTD) and S1-C-terminal domain (S1-CTD) [12]. The S2 consists of the fusion peptide (FP), heptad repeat 1 (HR1), and heptad repeat 2 (HR2); these structures function synchronously in the virus-host membrane fusion process for virus genome release into cytosol for replication [12]. At the N-terminal to the FP, there is an S2′, which is a furin cleavage site (shared by all members of coronavirus genera, [13]) to expose the FP that penetrates through the juxtaposed virus-host membranes [12]. More recently, structure and immune recognition of the PEDV S protein was studied [13]. Based on the HCoV NL63 S structure, the S1 subunit was divided into leader peptide sequence (residues 1–18), domains 0 (residues 19–219), A (residues 220–509), B (residues 510–639), and CD (residues 640–728) followed by S2 subunit which starts at residue 729 to the C-terminus [13,14]. Domain 0 (positioned beneath domain A) binds sialic acid on the host cell for virion attachment [15,16,17] and may also cause PEDV persistence and reoccurrence in the PEDV endemic pig farms [16]. Many B cell epitopes that induced production of neutralizing antibodies were found on domains A, B and CD of the S1 [14,18,19,20].

There is currently no direct-acting anti-PEDV drug. Vaccines against PEDV are not widely commercially available in North America, Europe, and some Asian countries, e.g., China, South Korea, Taiwan, and the Philippines [21]. The vaccine immunogens are either inactivated or live-attenuated viruses in the mono-, bi- or tri-valent format, recombinant proteins, DNA-vector or mRNA [21]. Multiple spaced doses of the vaccines are required to immunize sows or gilts either prefertilization or prefarrowing to induce high numbers of neutralizing antibodies in colostrum and milk that can confer passive immunity to suckling newborns. However, the immunization protocols of the vaccines are complicated and the protective efficacies among suckling piglets are inconsistent. Some studies have reported reduced morbidity, mortality, and quick recovery of daily weight gain of piglets born to vaccinated sows [22], while others did not find satisfactory reductions in morbidity rates and virus shedding among passively immunized piglets [23,24].

It is necessary that vaccines against coronaviruses including PEDV contain a whole array of neutralizing epitopes of surface exposed proteins, particularly those located in the S1 and S2 subunits. Regarding safety, the vaccine should not contain epitopes that elicit enhancing antibodies [25,26]. Several B cell epitopes of the PEDV S protein have been identified; they are either neutralizing epitopes with vaccine immunogen potential or are useful for diagnostic purposes [18,19,20,27,28,29,30,31]. In this study, a novel linear neutralizing B cell epitope of S1 protein, found commonly in PEDV G1 and G2 strains as well as other members of the *Alphacoronavirus* genus, was identified using PEDV neutralizing IgM monoclonal antibody as a biological tool. 

## 2. Materials and Methods

### 2.1. Ethics Statement of Animal Use

Mouse experiments were approved by Siriraj Animal Care and Use Committee, Faculty of Medicine Siriraj Hospital, Mahidol University, Bangkok, protocol number Si-ACUC no. 020/2561 (4 January 2019). The mice were housed in the Siriraj Laboratory Animal Research and Care Center. They were kept in individually ventilated cage with corncob and dry hyacinth bedding at 23 ± 1 °C, 12 h light/dark cycle, and 50–70% humidity. Feed pellets and drinking water were supplied ad libitum. Animal handling was performed by scientist holding a certificate for Laboratory Animal Use approved by the National Research Council of Thailand (NRCT). 

### 2.2. Cells and Virus Propagation

African green monkey kidney cells (Vero) and HeLa cells were from the American Type Culture Collection (ATCC, Manassas, VA, USA). They were maintained in Dulbecco’s modified Eagle’s medium (DMEM) (Thermo Fisher Scientific, Waltham, MA, USA) supplemented with 10% fetal bovine serum (FBS) (Sigma-Aldrich, St. Louis, MO, USA), 2 mM l-alanyl-l-glutaminase dipeptide, 100 IU/mL penicillin, and 100 µg/mL streptomycin (Thermo Fisher Scientific). Mouse SP2/0-Ag14 myeloma cells were maintained in RPMI-1640 medium (Thermo Fisher Scientific) supplemented similarly as for the complete DMEM. Cells were cultured at 37 °C in humidified 5% CO_2_ incubator. 

Porcine epidemic diarrhea virus P70 (field isolated G2 strain from infected piglet in Thailand) was propagated in Vero cells maintained in the virus culture medium DMEM supplemented with 2 µg/mL of *N*-tosyl-l-phenylalanine chloromethyl ketone (TPCK) treated-trypsin (Sigma-Aldrich) in T175 flasks. The cells at 80% confluent growth were washed twice with sterile phosphate buffered saline, pH 7.4 (PBS). PEDV virus was added to the cells at MOI 0.001 and incubated at 37 °C in humidified 5% CO_2_ incubator for 1 h to allow virus adsorption to cells. The extracellular fluid was removed; the cells were rinsed twice with sterile PBS and replenished with fresh virus culture medium. The infected cells were incubated further until maximal cytopathic effect (CPE) was revealed (approximately 48 h). The culture supernatant was harvested, and centrifuged at 4500× *g*, 4 °C for 30 min. The virus-containing culture supernatant was filtered through 0.2 µm Acrodisc^®^ syringe filter (Pall, Port Washington, NY, USA), aliquoted and kept at −80 °C until uses. The virus titer was determined by plaque assay and reported as plaque-forming units (pfu)/mL [20,32,33].

### 2.3. Production of Recombinant S1 Segment of PEDV Spike (S) Protein

Total RNA extracted from the PEDV P70 strain was used as a template for cDNA synthesis. The cDNA was synthesized using RevertAid First strand cDNA synthesis kit (Thermo Fisher Scientific). The gene sequence coding for S1 segment (*S1*; nt 1–2367) was PCR-amplified using the cDNA as the template, PCR primers (Table S1), and high fidelity Q5 DNA polymerase (New England Biolab, Ipswich, MA, USA). The PCR reaction mixture contained 10 µL of 5× Q5 reaction buffer; 1 µL of 10 mM dNTPs; 2.5 µL of *S1* specific forward primer; 2.5 µL of *S1* specific reverse primer; 2 µL of cDNA template (100 ng); 10 µL of 5× Q5 high GC enhancer; 0.5 µL of Q5 high-fidelity DNA polymerase; and 21.5 µL of molecular water. The PCR thermal cycles were 98 °C for 30 s (initial denaturation), 30 cycles of 98 °C for 30 s, 53 °C for 30 s and 72 °C for 2 min, and followed by final extension at 72 °C for 2 min. The *S1* amplicon was purified, subjected to A-tailing protocol to add the dA overhang, and cloned into pTZ57R/T (Thermo Fisher Scientific) via the dT overhang of the plasmid. The recombinant plasmids were introduced into competent DH5α *E. coli* by heat shock method. Selected clone of transformed DH5α *E. coli* positive for DNA insert of ~2500 bp was cultured in Luria-Bertani (LB) broth supplemented with 100 µg/mL ampicillin (LB-A) at 37 °C with shaking aeration overnight. The plasmids were extracted from the DH5α *E. coli* by using FavorPerp^TM^ plasmid extraction mini kit (Favorgen Biotech Corporation, Ping-Tung, Taiwan) and subcloned into pTriEx-1.1 Hygro DNA (Merck KGaA, Darmstadt, Germany) via *BamH*I and *Xho*I restriction sites. The recombinant *S1*-pTriEx1.1 plasmids were transformed into NiCo21 (DE3) *E. coli*. Bacterial transformant carrying the correct recombinant plasmid (verified by DNA sequencing) was grown in LB-A broth at 37 °C with shaking aeration to mid-log phase [optical density (OD) 600 nm was 0.4–0.5]. Expression of recombinant S1 protein (rS1) was induced by adding isopropyl β-d-1-thiogalactopyranoside (*IPTG*) to 0.1 mM final concentration, and the culture was incubated further for 3 h. The 8× His tagged-recombinant protein was purified from the bacterial homogenate using Ni-NTA affinity resin (Thermo Fisher Scientific) under denaturing condition with 8 M urea buffer. The purified recombinant protein was refolded slowly by stepwise dialysis with PBS, quantified by BCA assay, and verified by liquid chromatography-tandem mass spectrometry (LC-MS/MS). 

### 2.4. Preparation of Monoclonal Antibodies (mAbs) to the Recombinant S1 (rS1)

Female BALB/c mice, 6–8-weeks old, were immunized individually by intraperitoneal injection with 5 µg of rS1 protein in PBS mixed with Imject™ alum adjuvant (Pierce by Thermo Fisher Scientific) (3:1) (100 µL per mouse). The primed mice received two booster doses at 2 week-intervals. Seven days after the last booster, mice were bled and the presence of rS1 specific antibodies in their serum samples was determined by indirect ELISA. The immune mouse with the highest anti-rS1 serum antibody titer (1:820,000) and serum (1:10,000) revealed a reactive band to rS1 by Western blot analysis, and was given an intravenous booster with 5 µg of rS1 in 100 µL PBS. Three days later, the mouse was bled to collect immune serum (IS), and the animal was sacrificed humanely. The mouse single splenocytes were fused with log-phase grown-SP2/0-Ag14 myeloma cells at 5:1 ratio using 50% (*w*/*v*) polyethylene glycol 3350 as the cell fusogen. The fused cells were cultured in semisolid hypoxanthine-aminopterin-thymidine (HAT) selective medium (StemCell Technologies, Vancouver, Canada) at 37 °C in a humidified 5% CO_2_ incubator for 10 days. The hybridoma colonies were picked and nurtured in HT medium. Once the hybrids reached confluent growth, the spent culture fluids were harvested and checked for anti-rS1 antibodies by indirect ELISA. The clones that gave ELISA OD 405 nm to the rS1 higher than 0.35 were nurtured further in complete RPMI-1640 medium in six-well culture plates. The cell spent fluids of individual wells were evaluated for anti-rS1 antibody titer and binding activity to SDS-PAGE-separated rS1 by indirect ELISA and Western blot analysis, respectively. The hybridomas that gave high ELISA titers and their secreted mAbs reacted to the rS1 in Western blotting were expanded in serum-free medium for large-scale mAb production. The mAbs in the cell culture supernatants were isotyped using Rapid ELISA Mouse mAb Isotyping Kit (Thermo Fisher Scientific). The immunoglobulins were purified from the culture supernatants by using either HiTrap Protein G HP or HiTrap IgM HP (Cytiva, Uppsala, Sweden). Protein content of the purified mAbs were quantified by BCA assay (Thermo Fisher Scientific).

### 2.5. Enzyme-Linked Immunosorbent Assay (ELISA)

Individual wells of 96-well plates (Nunc MaxiSorp™, Thermo Fisher Scientific) were coated with a coating buffer (carbonate-bicarbonate buffer, pH 9.6) containing 100 µL of rS1 (3.25 µg/mL, optimal concentration from titration) and kept at 37 °C for 1 h. The empty sites of the well surface were blocked with 1% BSA in PBS containing 0.1% Tween-20 detergent (PBST). After washing with PBST, 100 µL of undiluted or two-fold serially diluted spent medium of the hybridomas or other mouse antibody preparations was added to appropriate antigen-coated wells, which were then incubated at 37 °C for 1 h. Horseradish-peroxidase (HRP) conjugated-goat anti-mouse immunoglobulin (Ig) antibody (Southern Biotech, Birmingham, AL, USA) (1:5000) was added and incubated at 37 °C for 1 h. After washing, ABTS substrate (KPL, Gaithersburg, MD, USA) was added and the plate was kept at room temperature (25 °C) in darkness for 30 min. The OD of each well content was measured at 405 nm against blank (reactants without mouse antibody) by spectrophotometry. 

### 2.6. Western Blot Analysis

Binding of the antibody preparations to the rS1 protein was determined by Western blotting. The rS1 (10 µg) in protein loading buffer was subjected to sodium dodecyl sulfate (SDS)-polyacrylamide gel electrophoresis (PAGE) (12% resolving gel cast in a buffer containing 1.5 M Tris-HCl, pH 8.8, and 4% stacking gel cast in 0.5 M Tris-HCl, pH 6.8 with 30% acrylamide/Bis solution (29:1) (Bio-Rad Laboratories, Hercules, California, USA), SDS, ammonium persulfate and *N*, *N*, *N*′, *N*′-tetramethylethylene-diamine (TEMED) (Bio-Rad Laboratories, Hercules, CA, USA). Electrophoresis was performed in electrode buffer at 20 mA constant current, until the font dye reached the lower gel edge. The SDS-PAGE-separated rS1 protein was electroblotted onto a nitrocellulose membrane (NC) and the empty sites on the membrane were blocked with 5% skim milk in Tris-buffered saline containing 0.1% Tween-20 (TBST). The membrane was cut along the direction of protein migration into strips (about 4 mm in width), and the strips were probed separately with culture fluids of individual hybridomas/antibody preparations to be tested. Buffer (PBS) and SP2/0-Ag14 myeloma spent medium served as negative binding control; mouse anti-His tag antibody (1:3000) (Bio-Rad Laboratories) and/or mouse immune serum (IS) to rS1 (1:10,000) were included as positive binding control(s). After incubation, the strips were washed with TBST, immersed in a solution of AP-conjugated-goat antimouse Ig antibody (1:3000) (Southern Biotech) in TBST, and kept at room temperature for 60 min. After washing the strips with TBST, BCIP/NBT substrate (KPL) was used to reveal the reactive bands. The reaction was stopped by rinsing the strips with distilled water.

### 2.7. Co-localization of the mAbs with S1 Overexpressed in Transfected-Mammalian Cells

HeLa cells were seeded onto a coverslip in a 24-well plate (1 × 10^5^ cells per coverslip) and incubated at 37 °C in a humidified, 5% CO_2_ incubator overnight. Cells were transfected with recombinant *S1*-pTriEx1.1 plasmids using a Lipofectamine™ 3000 transfection kit (Thermo Fisher Scientific) and incubated for 2 days to allow expression of the 8× His tagged-S1 protein. Thereafter, the cells were fixed with ice-cold 1:1 acetone-methanol for 10 min, rinsed with PBS and blocked with 5% FBS in PBS. Next, 10 µg of purified mAbs and rabbit anti-His antibody was added, and the samples were kept at room temperature for 1 h. After washing with PBS, goat anti-mouse IgG (H + L)-Alexa Fluor^®^ 488 (Thermo Fisher Scientific), goat anti-mouse IgM-Alexa Fluor^®^ 488 (BioLegend, San Diego, CA, USA), and donkey anti-rabbit IgG (H + L)-Alexa Fluor^®^ 555 (Invitrogen, Waltham, MA, USA) were applied as secondary antibodies. DAPI (Thermo Fisher Scientific) was used for locating nuclei. PBS served as a negative binding control.

### 2.8. Combined Co-Immunoprecipitation and Western Blotting

To determine the binding activity of mAbs to native S protein of PEDV, co-immunoprecipitation and Western blotting were performed. A Vero cell monolayer (100% confluent) in six-well plates was infected with a PEDV P70 strain at MOI 1.0 and incubated at 37 °C in humidified 5% CO_2_ incubator for 7 h. Infected cells were lysed by adding with M-Per^TM^ mammalian protein extraction reagent (Thermo Fisher Scientific) (400 µL/well) and incubated for 10 min. The infected cell lysate was clarified by centrifugation at 14,000× *g*, 4 °C for 15 min. The clear lysate was then absorbed sequentially with protein G followed by protein G coupled with mouse normal serum IgG, respectively, to remove nonspecific binding substances. Each 100-µL aliquot of the absorbed infected cell lysate was incubated with 10 µg of purified mAbs, or controls and the mixtures were kept at 4 °C with slow rocking for 3 h. Then, 15 µL of goat anti-mouse Ig sensitized-protein G was added to each reaction mixture, which was kept at 4 °C with rocking overnight, in order to pull down the immune complexes. Each reaction mixture was centrifuged at 500× *g*, 4 °C for 3 min; the immune complexes in the pellet were washed five times with PBS, resuspended with 50 µL of 1× protein-loading buffer, boiled for 5 min, and subjected to SDS-PAGE and Western blotting. The infected cell lysate alone (lysate) without any antibodies and the infected cell lysate with mouse immune serum to rS1 (IS) served as negative and positive controls, respectively.

### 2.9. PEDV Neutralization Assay

To determine the neutralizing activity of the rS1-bound mAbs, a neutralization assay was performed as described previously [20,21,22,23,24,25,26,27,28] with some modifications. Briefly, the purified mAbs were mixed with aliquots of PEDV P70 strain (100 pfu) and incubated at 37 °C for 1 h. The mixtures were then added to the wells of a 24-well tissue culture plate containing a confluent monolayer of Vero cells. Virus in medium alone was used as non-neutralization control. The preparations were incubated at 37 °C for 1 h to allow virus attachment to the cells. The free PEDV particles were removed, and the cells were washed twice with PBS. Carboxymethyl cellulose (CMC) (2% CMC in DMEM supplemented with 2 µg/mL of TPCK treated trypsin) was added to each well. The plate was incubated at 37 °C in a humidified, 5% CO_2_ incubator for 48 h. The cells were fixed with 10% formalin at room temperature for 1 h before staining with 1% crystal violet dye in 10% ethanol. The syncytial cells/foci were counted under light microscopy at 40× magnification. The percentage of mAb-mediated neutralization of PEDV infectivity was calculated: [1-(average CPE count of test/average CPE count of non-neutralization control)] × 100.

### 2.10. Epitope Mapping of the Neutralizing Monoclonal Antibody

The epitope of the PEDV neutralizing mAb was determined by means of phage mimotope identification and multiple S1 sequence alignments, and peptide-binding ELISA. 

For phage mimotope identification, the Ph.D.-12^TM^ phage display peptide library (New England Biolab, Ipswich, MA, USA) was used to identify the phage peptide that was bound by the neutralizing mAb (phage mimotope). Briefly, individual wells of a 96-well plate (Nunc MaxiSorp™, Thermo Fisher Scientific) were coated with 100 µL of the mAb in PBS and kept at 4 °C overnight. The empty sites on the well surface were blocked with 1% BSA in PBS and stored at 4 °C for 1 h. After washing with TBST, 100 µL of 2 × 10^11^ peptide-displaying phages from the library in 0.5% BSA-TBST was added to the mAb-coated wells, which were kept at room temperature for 1 h. The unbound phages were removed by washing and the mAb-bound phages were eluted with 100 µL of 0.2 M glycine-HCl, pH 2.2. The eluate was neutralized immediately by adding 15 µL of 1 M Tris-HCl, pH 9.1, inoculated into ER2738 *E. coli* culture to propagate the phages at 37 °C for 4.5 h. The bacterial culture was centrifuged and the phage particles in the supernatant were precipitated by adding 1/6 volume of 20% (*w*/*v*) PEG8000 and 2.5 M NaCl. The concentrated phage was titrated on LB/IPTG/Xgal plates and used for the second and third rounds of the panning. The eluted phages of the third panning round were diluted 10-fold serially (10^−1^–10^−4^) in LB broth, inoculated into mid-log phase grown ER2738 *E. coli*, mixed with prewarmed top agar and poured on LB/IPTG/Xgal plates. The plates were incubated at 37 °C overnight. Individual phage colonies on the agar were picked and inoculated into ER2738 *E. coli* for phage amplification. The culture supernatants were collected and clarified for phage DNA isolation and sequencing. The DNA sequences coding for peptides displayed by the phages (phage mimotopes) were deduced using QIAGEN CLC workbench program (Version 20.0.4) (https://digitalinsights.qiagen.com/ accessed on 25 November 2021). The phage mimotopic peptides were then multiply aligned with monomeric S1 sequences of CV777 classical strain (GenBank: AEX92968.1), PEDV P70 (strain of this study) and USA/Colorado/2013 (GenBank: AGO58924.1) by using Clustral Omega multiple sequence alignment in Jalview software (Version 2.11.1.6) [34]. A cryo-electron spike structure of PEDV (PDB: 6vv5) [13] was used to illustrate the mimotope that was bound by the PEDV neutralizing mAb. The structure was generated by using PyMOL software (Version 4.6) (The PyMOL Molecular Graphics System, Schrödinger LLC, New York, NY, USA).

To identify the neutralizing epitope of mAbA3 by peptide-binding ELISA, seven biotin-labeled overlapped peptides (12 amino acid in length with eight overlapped amino acids) that encompassed amino acid residues 240–275th of PEDV spike protein (base on PEDV P70 sequence) were synthesized (Genscript, Piscataway, NJ, USA). Individual peptides were diluted in PBS, added into individual wells of streptavidin-coated plate (Pierce™ Streptavidin Coated High Capacity Plates; Thermo Fisher Scientific) (1 µg in 100 µL PBS per well) and kept at 4 °C overnight. The empty sites on the well surface were blocked with 5% skim milk and kept at 37 °C for 1 h. After washing with TBST, 1 µg of mAb in 100 µL of TBST was added and samples were incubated at 37 °C for 1 h. Horseradish-peroxidase conjugated-goat anti-mouse immunoglobulin (Ig) antibody (Southern Biotech) (1:5000) was added and samples were further incubated at 37 °C for 1 h. After washing, ABTS substrate (KPL) was added into each well and the plate was kept at room temperature in darkness for 30 min. The OD of each well was measured at 405 nm by spectrometry. 

### 2.11. Multiple Sequence Alignment 

To determine whether the epitope of the PEDV neutralizing mAb was conserved among PEDV strains/genotypes and other members of *Alphacoronavirus* genus, the amino acid sequences of PEDV spike proteins of the PEDV causing recent outbreaks (published in Pubmed during the past 5 years) including JX-SCAU2020 (GenBank: QUE39276.1), CN/Liaoning/25/2018 (GenBank: QCW07226.1), POR-VC102 (GenBank: QQO86882.1), SF4017 (GenBank: QEQ55819.1), SP-VC3 (GenBank: QQO86847.1), and HNAY2016 (GenBank: QQS74747.1), canine coronavirus (CCoV) (GenBank: LC190907.1), feline coronavirus (FCoV) (GenBank: JN634064.1) and transmissible gastro-enteritis virus (TGEV) (GenBank: ABG89325.1) (Tables S2 and S3) were used for multiple alignments with the identified epitope sequence as described above.

### 2.12. Statistical Analysis

A statistical analysis was performed using the statistical software Graphpad Prism (Version 9.2) (San Diego, CA, USA). Data were analyzed by using one way ANOVA and were considered statistically significant when *p* < 0.05.

## 3. Results

### 3.1. Recombinant S1 of PEDV Spike Protein (rS1)

**Figure 1:**
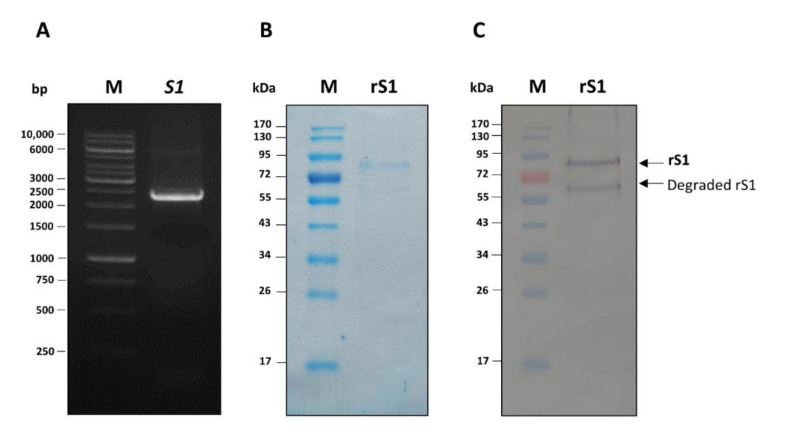
Production of recombinant S1 (rS1) of porcine epidemic diarrhea virus (PEDV). (**A**) Polymerase chain reaction (PCR) amplificon of PEDV S1 gene (*S1*). Lane M, 1 kilobase (kb) DNA ladder. Numbers on the left are DNA sizes in base pairs (bp). (**B**) Sodium dodecyl sulfate-polyacrylamide gel electrophoresis (SDS-PAGE) separated pattern of the purified rS1 protein after staining with Coomassie Brilliant Blue G-250 (CBB) dye. (**C**) Western blot pattern of the rS1 as detected by anti-His antibody. Lanes M and numbers on the left of (**B**) and (**C**) are protein molecular mass marker and protein molecular masses in kilo-Daltons (kDa), respectively.

DNA coding for PEDV S1 (*S1*, nt 1–2367) was successfully amplified (Figure 1A). The recombinant protein produced by the *S1*-pTriEx1.1 transformed-NiCo21(DE3) *E. coli*, purified using Ni-NTA affinity resin under denaturing conditions and refolded, was analyzed by SDS-PAGE and with CBB staining and Western blotting. The results are shown in Figure 1B,C, respectively. The purified recombinant protein at ~89 kDa was verified as PDEV S1 protein by LC-MS/MS (data not shown).

### 3.2. Hybridoma Generation and Production of mAbs to Recombinant S1 Protein

**Figure 2:**
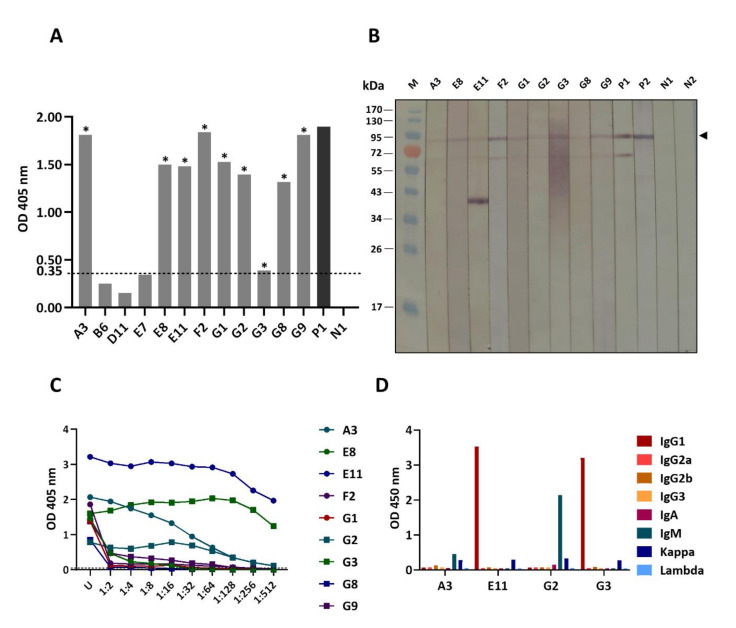
Binding of monoclonal antibodies (mAbs) in culture fluids of different hybridoma clones to rS1 and titers and isotypes of the mAbs secreted by the selected hybridomas. (**A**) Indirect ELISA OD 405 nm of mAbs in individual hybridoma culture fluids to the rS1. *, the clone that gave OD 405 nm ≥ 0.35 (arbitrarily cut-off level), which was selected for further testing. P1, mouse immune serum (1:50,000) to rS1 (IS) as positive binding control; N1, SP2/0-Ag14 spent medium as negative binding control. Broken line, cut-off OD. (**B**) Binding of the mAbs of the selected hybridoma clones to SDS-PAGE-separated rS1 (arrowhead) by Western blotting. P1 is mouse immune serum to rS1 (IS) (1:10,000) and P2 is anti-His antibody, both served as positive controls for binding to 8× His tagged-rS1. N1 and N2 are SP2/0-Ag14 spent medium and PBS, respectively, which served as negative binding controls. Lane M, protein molecular mass marker. Numbers on the left are protein molecular masses in kDa. The prominent band at about 40 kDa (lane E11) should be N-terminal portion of degraded rS1 which contained epitope of the mAbE11; this band was not bound by the anti-His (P2) as the recombinant S1 had the 8× His tag at the C-terminus; the band was not recognized by the mouse immune serum (diluted 1:10,000) to rS1, as the antibody to this N-terminal epitope might be diluted out. (**C**) Indirect ELISA titers of the rS1-specific mAbs secreted from hybridoma clones. Monoclonal antibodies of the clones G2, G3, A3, and E11 clones yielded high titers at the maximal phase of cell growth; these clones were selected for further experiments. (**D**) Isotype identification of the mAbs of the selected A3, G2, G3, and E11 clones.

The mouse immune splenocytes (8.5 × 10^7^ cells) were fused with SP2/0-Ag14 myeloma cells (approximately 5:1), and the fused cells were grown in HAT selective medium. The spent culture fluids of the growing hybridomas were screened for rS1 antibodies by indirect ELISA, and the culture fluids of nine clones (A3, E8, E11, F2, G1, G2, G3, G8 and G9) gave significant OD 405 nm to the rS1 (cut-off level was arbitrarily set at OD 405 nm ≥ 0.35) (Figure 2A). Monoclonal antibodies of these clones also bound to SDS-PAGE-separated rS1 (~89 kDa) by Western blot analysis (Figure 2B). At the maximal phase of the cell growth, four hybridoma clones (A3, E11, G2 and G3) gave ELISA titer against rS1 equal to/or higher than 512 (Figure 2C). These clones were selected for further experiments. The mAbs of clones A3 and G2 belong to IgM isotype, while those of clones G3 and E11 are IgG1 isotype. Monoclonal antibodies of all four clones carry kappa light chains (Figure 2D). 

### 3.3. Colocalization of mAbs to PEDV S1 Overexpressed in Transfected-HeLa Cells

**Figure 3:**
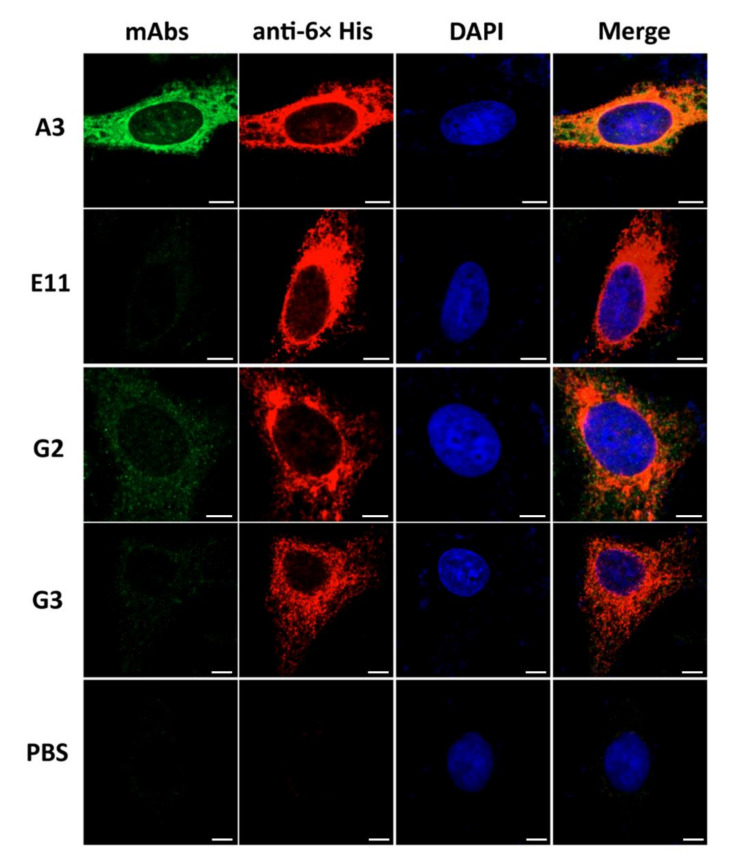
Co-localization of the rS1-specific-mAbs to PEDV S1 overexpressed in HeLa cells as determined by confocal microscopy. HeLa cells transfected with *S1*-pTriEx 1.1 recombinant plasmid were cultured on cover slips in 24-well culture plate for 48 h. Cells were fixed with cold acetone-methanol (1:1), followed by staining with 10 µg of individual mAbs to S1 (anti-rS1) and rabbit anti-His antibody (1:500) as primary antibodies and fluorophore-conjugated secondary antibodies (1:300). mAbA3, mAbG2 and mAbG3 (green) colocalized with intracellular S1 overexpressed in the HeLa cells (red) and seen as orange or yellow matter in merge. The mAbE11 did not bind (or bound negligibly) to the overexpressed S1 in the transfected cells. Nuclei stained blue by DAPI dye. Scale bar = 5 µm.

Confocal microscopy was used to determine colocalization of the mAbs with the intracellularly produced S1 in mammalian cells. Figure 3 demonstrates the colocalization (orange/yellow) of mAbA3, mAbG2 and mAbG3 (green) with S1 (red) in the PEDV-S1 transfected HeLa cells. Colocalization of the mAbE11 with the intracellular S1 was negligible (if it occurred at all).

### 3.4. Binding of the mAb to Native S Protein

**Figure 4:**
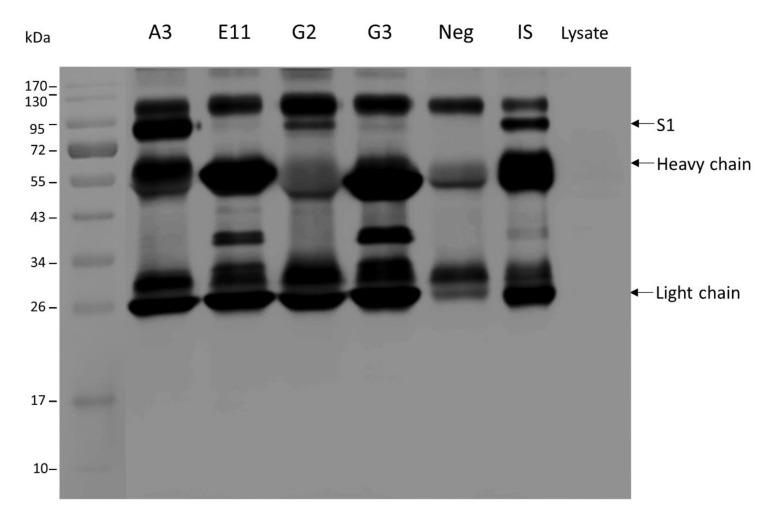
Binding of mAbs to native S1 protein. Combined co-immunoprecipitation and Western blotting was conducted to determine the binding activity of mAbs to native spike protein of PEDV. Ten micrograms of mAbs of individual hybridoma clones were mixed with lysate of PEDV infected cells. The mAb-Ag complexes were pull-down by using goat antimouse Ig sensitized-protein G. The pull-down content was then subjected to Western blotting by probing the SDS-PAGE-separated components with mouse immune serum against the PEDV rS1 (IS). The mAbA3, mAbG2, and mAbG3 could bind to the native S1 protein contained in the PEDV infected cell lysate and were pull-down together as demonstrated, i.e., the presence of the S1 band (~90 kDa, upper arrow) and the bands of heavy chains (~50–70 kDa, middle arrow) and light chains (~25 kDa, lower arrow) of the mAbs in Western blot analysis. Neg, reaction without mAb; IS, reaction added with mouse immune serum to PEDV rS1; Lysate, reaction containing PEDV infected cell lysate alone without any antibody.

Co-immunoprecipitation and Western blotting were used to demonstrate mAb binding to the native PEDV S protein prior to testing the mAb in the PEDV neutralization assay. Ten micrograms of mAbs were mixed with the absorbed PEDV infected cell lysate. The mAb-Ag complexes were pulled down by adding with goat antimouse Ig sensitized-protein-G and analyzed by Western blot analysis. The reactive bands on SDS-PAGE were determined by LC-MS/MS (data not shown). Monoclonal antibodies of clone A3 (mAbA3), mAbG2 and mAbG3 coprecipitated with the native spike protein of the PEDV (~90 kDa), as demonstrated by Western blot analysis. The SDS-PAGE-separated immune complexes were probed with mouse immune serum to rS1 (IS) (Figure 4). A faint S1 band was revealed in the mixture of the PEDV-infected cell lysate with the mAbE11.

### 3.5. Neutralization of PEDV Infectivity by the S1 mAb

**Figure 5:**
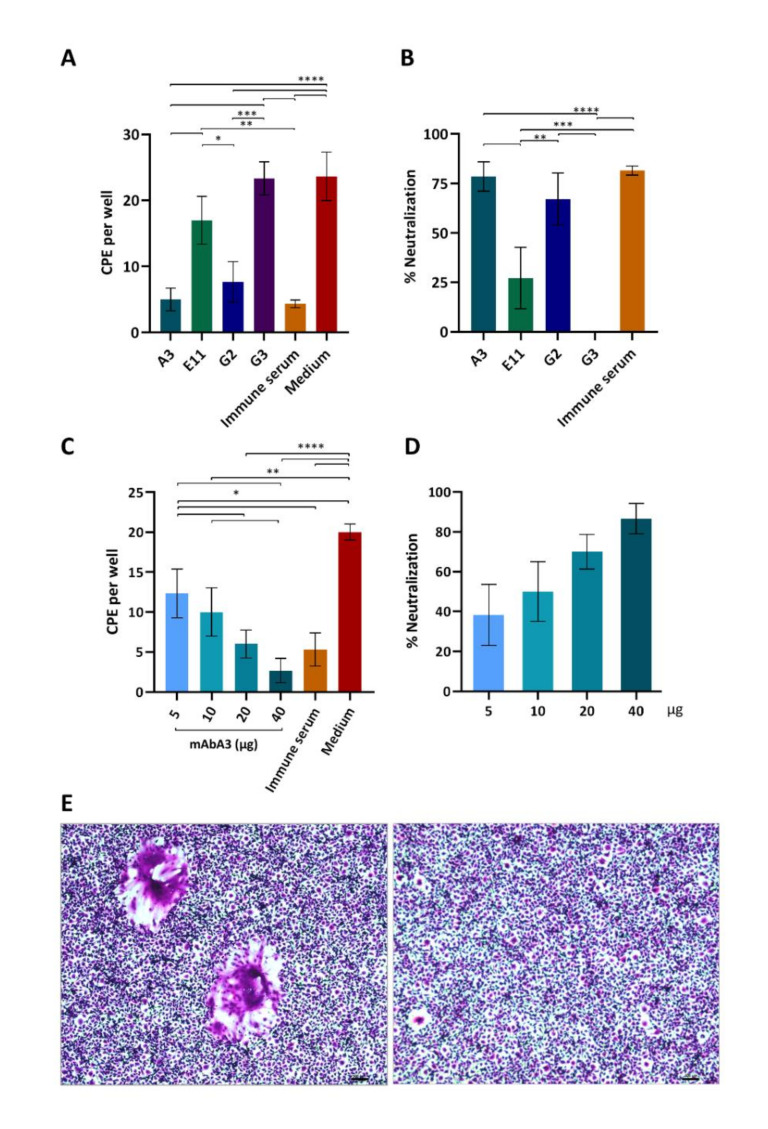
Monoclonal antibody A3-mediated neutralization of PEDV infectivity. (**A**) Preliminary experiments using 10 µg of mAbA3, mAbE11, mAbG2 and mAbG3 to mix with 100 pfu of PEDV P70 G2 strain before adding to the Vero cell monolayer. Infected cells treated with mouse immune serum to rS1 (IS) and infected cells in medium alone served as positive and negative neutralization controls, respectively. After 1 h, the fluid in each well was removed; the cells were washed and grown in semisolid virus maintaining medium for 48 h. Then, the cells were fixed with 10% formalin and stained with 1% crystal violet dye. The CPE (syncytial cells) were counted under 40× magnification light microscopy. Mean and standard deviation of the CPE number in triplicate wells of each treatment are reported. (**B**) Percent PEDV neutralization mediated by mAbs of individual clones and IS. (**C**) PEDV (100 pfu) was mixed with indicated amount of purified mAbA3 (5–40 µg) before adding to monolayer of Vero cells in 24-well culture plate. Mouse immune serum (IS, 1:200) and the virus in medium alone were used as positive and negative neutralization controls, respectively. Numbers of CPE per well (mean ± SD of triplicate wells) are shown. (**D**) Percentage of mAbA3 mediated neutralization of PEDV infectivity compared to infected cells in medium alone. Three replicative experiments were performed. *, *p* < 0.05; **, *p* < 0.01; ***, *p* < 0.001; ****, *p* < 0.0001 by one-way ANOVA. (**E**) Appearance of syncytial Vero cells (left) and normal Vero cells (right). Scale bar = 100 nm.

Preliminary experiments using 10 µg of mAbA3, mAbE11, mAbG2 and mAbG3 to mix with 100 pfu of the PEDV P70 G2 strain before adding to the Vero cell monolayer indicated that mAbA3 (IgM isotype) could significantly reduce the cytopathic effect (CPE) of the PEDV compared to the Vero cells infected with the virus in medium alone (Figure 5A,B). Therefore, this clone was produced on a large scale and retested at different concentrations (5, 10, 20, and 40 µg) for neutralization of the PEDV infectivity. Figure 5C shows numbers of CPE [mean ± standard deviation (SD)] of individual treatments. Percentages of CPE reduction mediated by the mAbA3 were dose-dependent (Figure 5D). Three replicative experiments were performed, and data of a representative experiment are shown. Figure 5E illustrates examples of CPE (syncytial formation) of PEDV infected Vero cells compared with noninfected cells.

### 3.6. Epitope Mapping by Means of Phage Mimotope Identification

**Figure 6:**
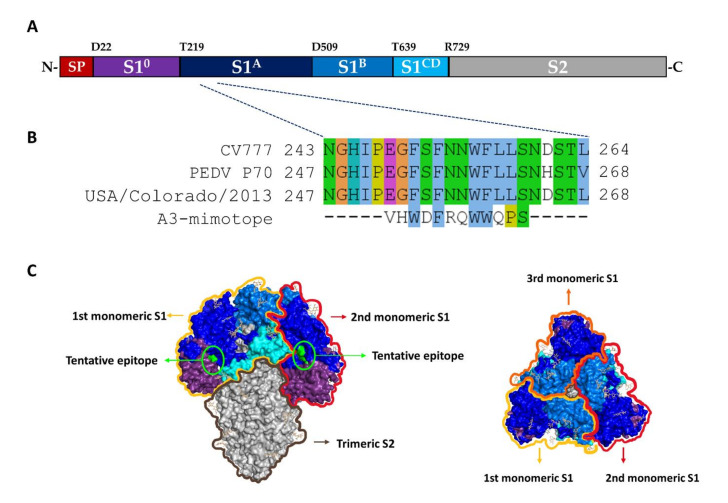
Presumptive amino acid sequence that was bound by mAbA3 as determined by phage mimotope identification using Ph.D.^TM^-12 Phage display peptide library. (**A**) Domain organization of spike protein of PEDV P70 strain (adapted from [25]). S1 portion can be divided into 4 subdomains including S1^0^ (purple); S1A (dark blue); S1B (marine); and S1C and D (cyan). (**B**) Amino acid sequence of phage mimotope that was bound by mAbA3 (A3-mimotope). Multiple sequence alignment of A3-mimotope with S1 segments of CV777 classical strain (GenBank: AEX92968.1), PEDV P70 (strain of this study) and USA/Colorado/2013 (GenBank: AGO58924.1; the strain that Cryo-EM structure is available, PDB: 6vv5) revealed that the tentative epitope “252EGFSFNNWFLLS263” is located at the S1A subdomain. Each amino acid was colored by Clustal X scheme, which is based on chemical characteristic of amino acid. (**C**) Illustration of tentative epitope of the mAbA3 (green) on the PEDV spike protein homotrimer: side view (left) and top view (right).

The domain organization of the PEDV spike protein is shown in Figure 6A (adapted from Li et al. 2017, [14]). The Ph.D.^TM^-12 phage display peptide library was used to identify the amino acid sequence of phage peptide that was bound by the mAbA3. After three rounds of phage peptide bio-panning with the mAbA3, 10 individual phage clones were randomly picked for phage DNA extraction and sequencing. Deduced amino acid sequences of all 10 phage clones revealed identical peptide, i.e., “VHWDFRQWWQPS”, designated “A3-mimotope” (Figure 6A). The A3-mimotopic peptide was aligned with monomeric S1 of CV777 which is a PEDV classical strain (GenBank: AEX92968.1), PEDV P70 strain (this study) and USA/Colorado/2013 (GenBank: AGO58924.1) by using Clustral omega multiple sequence alignment for identification of tentative S1 residues bound by mAbA3. The result matched with “252EGFSFNNWFLL**S**263” located in S1A subdomain of PEDV (Figure 6B). Figure 6C Illustrates tentative location of the mAbA3 epitope on the PEDV spike protein homotrimer.

### 3.7. Peptide-Binding ELISA for mAbA3 Epitope Validation

**Figure 7:**
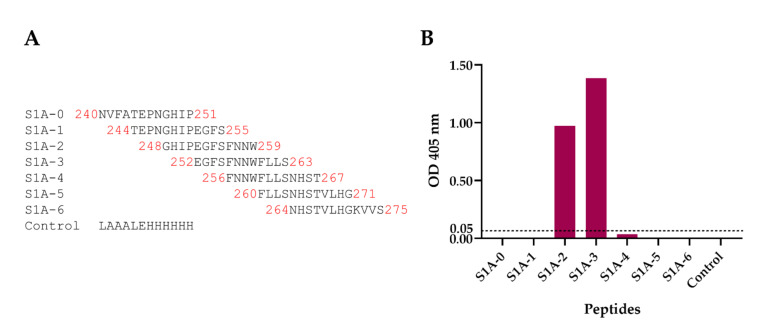
Overlapped peptides of PEDV spike protein bound by the mAbA3 as tested by peptide-binding ELISA. Biotin-labeled 12-mer overlapped peptides encompass PEDV spike protein (amino acid residues 240–275th) were used in the indirect ELISA for testing mAbA3 binding. (**A**) List and amino acid sequences of the 8 overlapped peptides of the PEDV spike protein. (**B**) OD 405 nm of mAbA3 that bound to individual overlapped S1 peptides and control peptide. The mAbA3 bound to peptides S1A-2 (248GHIPEGFSFNNW259) and S1A-3 (252EGFSFNNWFLLS263) and yielded high ELISA signals.

Biotin-labeled, overlapped peptides (12 amino acids in length with eight overlapped amino acids) encompassing the 240th–275th residues of the PEDV spike protein were synthesized (Figure 7A) and used in the indirect ELISA (using streptavidin-coated plate) to confirm the tentative S1 residues and region that were bound by the mAbA3. As shown in Figure 7B, the mAbA3 bound to peptides S1A-2 (248GHIPEGFSFNNW259) and S1A-3 (252EGFSFNNWFLLS263) with high binding signals. The indirect ELISA results matched with the tentative S1 residues identified by phage mimotope (A3-mimotope, 252EGFSFNNWFLLS263). The peptide ELISA results indicated that the PEDV neutralizing epitope of the mAbA3 (A3-epitope) is a linear sequence located in the S1A subdomain of the PEDV S protein.

### 3.8. The Novel A3-Epitope Is Shared among Alphacoronaviruses

**Figure 8:**
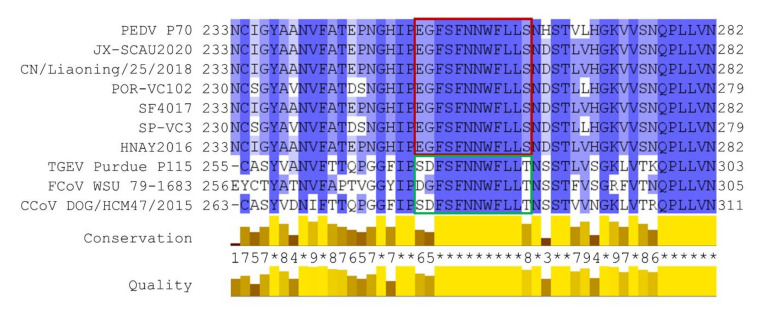
Multiple alignments of the amino acid sequences of spike proteins of various strains of PEDV that were published in PubMed during the past 5 years and other alphacoronaviruses. The amino acid sequences of PEDV that caused PED outbreaks included JX-SCAU2020 (GenBank: QUE39276.1); CN/Liaoning/25/2018 (GenBank: QCW07226.1); POR-VC102 (GenBank: QQO86882.1); SF4017 (GenBank: QEQ55819.1); SP-VC3 (GenBank: QQO86847.1); and HNAY2016 (GenBank: QQS74747.1). The sequences of other alphacoronaviruses include TGEV Purdue P115 (GenBank: ABG89325.1); FCoV WSU 79-1683 (GenBank: JN634064.1); and CCoV DOG/HCM47/2015 (GenBank: LC190907.1). The multiple sequence alignment revealed that the A3-epitope that was identified from the phage mimotope and overlapped S1 peptide-binding ELISAs, “EGFSFNNWFLLS”, is identical among the PEDV spike sequences (red box) and this sequence is found to be highly conserved among the other alphacoronaviruses (green box).

To determine whether the A3-epitope identified from this study was conserved across the PEDV strains and genotypes and other members of the *Alphacoronavirus* genus, the amino acid sequences of PEDV spike proteins of PEDV isolates that caused recent outbreaks published in PubMed during the past 5 years and other alphacoronaviruses were multiply aligned with the A3-epitope. As shown in Figure 8, the A3-epitope was identical among the PEDV isolates across G1 and G2 genotypes (red box). The epitope is also shared (highly conserved) among the alphacoronaviruses (green box).

## 4. Discussion

PEDV can infect pigs of all ages, although only newborn piglets succumb to severe illness [4,35]. Once an outbreak has occurred in a farm (of either the farrowing-weaning or farrowing-growing-finishing production types), PEDV may remain in circulation due to its persistence in asymptomatically or subclinically infected pigs, from which it can be transmitted to susceptible newborns [36,37]. The vaccination of sows or gilts against PEDV is the current strategic mainstay to cope with infections among suckling piglets. The rationale is to confer passively-acquired lactogenic immunity upon newborns until they reach an age at which they are no longer susceptible to severe symptoms. Although vaccines against PEDV exist, their availability is limited in certain countries. Current PEDV vaccines (in use or developing) are either the traditional, first-generation vaccines consisting of live attenuated or inactivated whole PEDV [38,39], second-generation vaccines composed of either the recombinant virus protein [40] or virus-like particles (VLP) [41] combined with immunological adjuvant, or third-generation vaccines comprising DNA plasmid carried by a vector capable of expressing the PEDV protein antigen in the host [42,43]. However, vaccine efficacy is unsatisfactory and recurrent PEDV outbreaks occur [44,45]. Moreover, immunization with some PEDV vaccines has resulted in enhancement of disease symptoms and virus replication in vaccinated pigs exposed to PEDV challenge [46]. There is still a need for a more effective and safe vaccine against the disease. 

Regarding vaccine ontogeny, significant advantages have been observed in using a synthetic peptide containing only neutralizing epitope(s) over whole molecule of the cognate protein [43,47]. Usually, the surface exposed spike glycoproteins (S) of coronaviruses play a pivotal role in cell attachment, host cell receptor binding, and virus internalization for further replication [4,48,49]. Therefore, the S proteins are the main targets of vaccines against coronaviruses, as they can induce antibodies that neutralize infectivity [14,18,19,20]. Nevertheless, several coronavirus vaccines that contain either whole viruses (live attenuated or inactivated), adjuvanted recombinant spike protein/VLP, or plasmid DNA or mRNA coding for the S protein, have been shown to inflict different degrees of adverse effects on vaccinated subjects/animals. One matter of particular concern in vaccination (as well as passive immunization using post-vaccinated or convalescing plasma antibodies) against virus infections is antibody-dependent enhancement (ADE). For respiratory virus infections, the exacerbated diseases often observed after vaccination mediated by ADE is termed “Vaccine associated enhanced respiratory disease (ERD)” [50]. Currently, at least four ADE mechanisms have been elucidated: (1) increment of virus entry into mononuclear phagocytes via the Fc receptors, causing increment of the virus load (extrinsic ADE) [51,52]; (2) immune complex-mediated-inflammation through complement activation and recruitment of inflammatory and immune cells (ADE via enhanced immune activation) [53]; (3) the infecting virus inhibits type 1 interferon and activation of interleukin-10 biosynthesis, thereby favoring a Th2 type immune response, which heightens the virus production (intrinsic ADE) [51]; and (4) the antibody enhances the virus entry into permissive cells. In the case of coronavirus infections, the enhancing antibody promotes the up-standing form of the receptor binding domain [25], while in influenza virus infection, the ADE occurs via the promotion of hemagglutinin stem loop flexibility and virus fusion kinetics [35]. For PEDV immunization, one study observed that piglets immunized with PEDV S protein derived from standard Bac-to-Bac Baculovirus expression system developed high levels of PEDV-neutralizing antibodies; nevertheless, the vaccinated pigs suffered exacerbated clinical diarrhea and disease following homologous PEDV challenge [46]. Vaccine-induced enhancement of coronavirus replication and disease were also observed for feline infectious peritonitis [54] and human severe acute respiratory syndrome [55]. Thus, for safety reasons, next generation vaccines should elicit broadly neutralizing antibodies and not enhancing antibodies (i.e., the vaccine should contain only virus neutralizing epitopes). For PEDV vaccines, multiple neutralizing epitopes of the PEDV S1 with vaccine potential have been identified [18,20,27,29,56,57,58,59].

In this study, hybridomas secreting mAbs that neutralized the infectivity of the field isolated PEDV strain of genotype 2 that caused a PED outbreak in Thailand were generated. One hybridoma clone, A3, that grew well in serum-free culture medium produced IgM mAb (mAbA3) that not only bound to the homologous antigen, i.e., bacterially derived recombinant S1 protein, and the native counterpart in the PEDV *S1* transfected- and PEDV infected cells, but also neutralized the infectivity of the PEDV strain, as determined by a cell-based neutralization assay. Therefore, mAbA3 has potential for use in the passive immunization of PEDV susceptible piglets, particularly those born to nonimmune mothers. Moreover, the mAbA3 neutralizing epitope, along with the previously identified PEDV spike protein neutralizing epitopes, may serve as an immunogenic cocktail for a safe and effective next generation PEDV vaccine. 

By means of a phage mimotope searching, the mAbA3 was found to bind to phages that carried “VHWDFRQWWQPS” peptide. After the multiple sequence alignment with the amino acid sequences of S1 of various PEDV strains, i.e., PEDV P70 (G2 field isolated strain), PEDV CV777 (classical strain), and PEDV USA/Colorado/2013, the mimotope was found to match with “252EGFSFNNWFLLS263” of the S1A subdomain of the three PEDV strains. The tentative mAbA3 epitope was verified by means of peptide-binding-indirect ELISA using streptavidin-coated plate to capture biotin on each peptide. The results showed that the mAbA3 bound to the overlapped peptides S1A-2 “248GHIPEGFSFNNW259” and S1A-3 “252EGFSFNNWFLLS263” of the S1A subdomain of the S protein, which involved in host cell attachment. One previous study reported a neutralizing epitope located on residues 435–485 of the PEDV S1A subdomain. However, mAbs directed against that epitope was shown to neutralize the infectivity of only G2b, and not the G1 PEDV classical strain CV777 [29,57]. This might have been due to the critical amino acid substitutions that occurred in the residues 435–485 region of the G2b variant that were different from those of the CV777 classical G1 strain. Our neutralizing mAbA3 bound to a novel linear epitope of the S1A subdomain of the S protein. From multiple sequence alignment, the mAbA3-epitope is shared by both G1 and G2 PEDV strains and other alphacoronaviruses, including canine- and feline-coronaviruses and porcine transmissible gastroenteritis virus (TGEV), indicating that this region of the S1 protein is highly conserved and could be indispensable for alphacoronaviruses during initial infection. Unfortunately, heterologous coronaviruses are not currently available for laboratory testing of the mAbA3 neutralizing activity. Nevertheless, the overall data reported herein indicate that mAbA3 has potential as a ready-to-use antibody for passive immunization of PEDV susceptible piglets. Also, inclusion of the mAbA3-epitope in a PEDV vaccine made of a cocktail of PEDV neutralizing epitopes (and cell-mediated epitopes) would likely not only increase the vaccine’s protective efficacy against the PEDV, but might also elicit cross-protection against other alphacoronaviruses, particularly porcine TGEV, thereby killing two (or more) birds with one stone.

## 5. Conclusions

In this study, a novel, linear neutralizing epitope of the PEDV spike (S) protein was identified using mouse IgM monoclonal antibodies (mAbA3) against the recombinant S1 of PEDV G2 strain. The mAb epitope (A3-epitope) is located on the S1A subdomain of the S protein, which is important for receptor binding for cellular entry. The epitope is shared by the PEDV G1 and G2 strains, as well as other alphacoronaviruses including canine- and feline-coronaviruses and porcine transmissible gastroenteritis virus. A subunit PEDV vaccine containing this epitope should be protective not only against the PEDV, but also against other alphacoronavirus infections. As such, mAbA3 has potential for use in the passive immunization of susceptible piglets against PEDV.

## Data Availability

All datasets presented in this study are included in the article and Supplementary Materials.

## Supplementary Materials

The following are available online at https://www.mdpi.com/article/10.3390/v14010125/s1. Supplementary Table S1: Oligonucleotide primers used in this study for production of recombinant S1 subunit of PEDV spike protein; Table S2: PEDV that caused recent outbreaks; Table S3: Other alphacoronaviruses. References [44,60,61,62,63] are cited in the supplementary materials.
