# Supplementary material for: Novel Neutralizing Epitope of PEDV S1 Protein Identified by IgM Monoclonal Antibody
Source: Viruses. 2022 Jan 11;14(1):125. doi: 10.3390/v14010125 (PMC8778753; doi:10.3390/v14010125)
Supplement: Supplementary file 1 [file viruses-14-00125-s001.zip › viruses-1517412-supplementary.pdf]

viruses

MDPI

Supplementary materials

Novel Neutralizing Epitope of PEDV S1 Protein Identified by IgM Monoclonal Antibody

Table S1. Oligonucleotide primers used in this study for production of recombinant S1 subunit of PEDV spike protein.

Genes	Primer	Sequence
S1	PEDV_S1 F′	5′-GCGGATCCGATGAARTCYTTAAMYTACTTCTGG-3′
	PEDV_S1 R′	5′-GGGCTCGAGTGAACCGCCTCCACCRTGGTARAAGAAAC-CAGG-3′

Table S2. PEDV that caused recent outbreaks and their S1 sequences were used in this study for multiple alignments.

No.	Isolate name	Isolated year	Country	Genotype	References
1	JX-SCAU2020	2020	China	-	[44]
2	CN/Liaoning/25/2018	2019	China	-	[60]
3	POR-VC102	2019	Portugal	G1b	[61]
4	SF4017	2017	Philippines	G2	[62]
5	SP-VC3	2017	Spain	G1b	[61]
6	HNAY2016	2016	China	-	[63]

-, not available.

Table S3. Other alphacoronaviruses that their S1 sequences were used in this study for multiple alignments **.**

Virus name	Isolate name	Isolated year	Country	GenBank no.
Transmissible gastro-enteritis virus (TGEV)	Purdue P115	2006	United states of America	ABG89325.1
Feline coronavirus (FCoV)	WSU 79/1683	2011	United states of America	JN634064.1
Canine coronavirus (CCoV)	DOG/HCM47/201 5	2015	Vietnam	LC190907.1
